# Fruit and Vegetable Consumption among Immigrants in Portugal: A Nationwide Cross-Sectional Study

**DOI:** 10.3390/ijerph15102299

**Published:** 2018-10-19

**Authors:** Liliane Costa, Sónia Dias, Maria do Rosário O. Martins

**Affiliations:** 1 Global Health and Tropical Medicine, GHTM, Instituto de Higiene e Medicina Tropical, IHMT, Universidade Nova de Lisboa, UNL, Rua da Junqueira 100, 1349-008 Lisboa, Portugal; mrfom@ihmt.unl.pt; 2 Escola Nacional de Saúde Pública, Centro de Investigação em Saúde Pública, Universidade NOVA de Lisboa & Global Health and Tropical Medicine, GHTM, Instituto de Higiene e Medicina Tropical, Universidade Nova de Lisboa, 1600-560 Lisboa, Portugal; sonia.dias@ensp.unl.pt

**Keywords:** fruit, vegetable, immigrant, Portuguese, health

## Abstract

This study aims to compare adequate fruit and vegetable (F&V) intake between immigrants and natives in Portugal, and to analyse factors associated with consumption of F&V among immigrants. Data from a population based cross-sectional study (2014) was used. The final sample comprised 17,410 participants (≥20 years old), of whom 7.4% were immigrants. Chi-squared tests and logistic regression models were conducted to investigate the association between adequate F&V intake, sociodemographic, anthropometric, and lifestyle characteristics. Adequate F&V intake was more prevalent among immigrants (21.1% (95% CI: 19.0–23.4)) than natives (18.5% (95% CI: 17.9–19.1)), (*p* = 0.000). Association between migrant status and adequate F&V intake was only evident for men: immigrants were less likely to achieve an adequate F&V intake (OR = 0.67, 95% CI = 0.66–0.68) when compared to Portuguese. Among immigrants, being female, older, with a higher education, and living in a low urbanisation area increased the odds of having F&V consumption closer to the recommendations. Adjusting for other factors, length of residence appears as a risk factor (15 or more years vs. 0–9 years: OR = 0.52, 95% CI = 0.50–0.53), (*p* = 0.000) for adequate F&V intake. Policies aiming to promote adequate F&V consumption should consider both populations groups, and gender-based strategies should address proper sociodemographic, anthropometric, and lifestyle determinants.

## 1. Introduction

Fruit and vegetables are important components of a healthy diet and could help prevent major diseases, such as cardiovascular diseases, obesity, type 2 diabetes, and certain cancers, especially if integrated in an active and healthy lifestyle [1,2]. Therefore, fruit and vegetable (F&V) consumption is used worldwide as an important diet quality indicator [3]. F&V are rich sources of dietary fibre, vitamins, minerals, and phytochemicals, which may explain these positive effects on health [4]. A prospective cohort study conducted during 7.4 years, in 18 low-income, middle-income, and high-income countries from the five continents, among 135,335 individuals, showed that total F&V intake was associated with lower noncardiovascular mortality and total mortality, in a multivariable adjusted model [5,6]. The risk estimate for all-cause mortality, cardiovascular disease, and cancer, decreases by 10% and 11%, with consumption above 250–300 grams per day (g/d) of fruit and 300 g/d of vegetables, respectively [6]. According to World Health Organization (WHO), an adequate quantity of F&V of 400 to 500 g/d or five portions is recommended [1,2]. Worldwide, mean intakes of F&V were below current recommendations, and higher consumption was observed among women and older adults [7]. The National Food and Physical Activity Survey 2015–2016 (IAN-AF) revealed that one in two Portuguese do not consume the quantities of F&V recommended by the WHO [8].

Migration is a worldwide phenomenon and may contribute as an important experimental framework for investigate the effects of environmental factors on diet and health disparities. According to the Statistical Office of the European Communities (EUROSTAT), at the beginning of 2016, there were 35.1 million immigrants living in a European Union (EU) member state that were born outside of the EU-28, and 19.3 million were born in a different EU member state [9]. In Portugal, between 1995 and 2013 foreign-born immigrants rose from 5.2% to 8.2% of the population [10].

Migration has been associated with dietary changes, although generalisations must be carefully taken due to different individual, social, and economic factors affecting immigrants before and after arriving in the new country [11,12,13]. When arriving at the host country, immigrants may retain, adapt, or exclude traditional foods, and adopt the eating patterns/food choices in part or in whole, through a process called “dietary acculturation” [14]. This is a multidimensional and dynamic process that may have either positive or negative implications for health. Length of residence in the host country is often a measure of acculturation, and has been reported as an important determinant of immigrants’ health [15,16]. In the literature, dietary intake has been associated with length of residence, with mixed benefits [17]. However, in general, detrimental effects on diet have been observed among immigrants and racial/ ethnic minorities [18,19].

Immigration has known to have a strong impact on dietary practices and also on health disparities among immigrants and between minority groups and natives [13,19,20]. Therefore studies among foreign-born and comparisons with natives would likely be of interest for disparities research. Most studies on adult immigrants’ frequency and determinants of F&V intake were conducted in the United States of America (USA) mostly among racial and ethnic groups [21,22,23,24]. In Canada, studies between immigrants and natives concluded that more information would be needed about nutritional health and immigration-related dietary changes [15]. In Europe, few studies are available on adequate F&V consumption and they mainly focus on immigrants from low- and middle-income countries [25,26,27]. In Portugal, scarce information is available on frequency, distribution, and determinants of adequate F&V consumption among adults and immigrants, and little is known about differences between native and immigrant food habits.

A more comprehensive understanding on adherence of F&V intake to dietary recommendations, and factors that influence its consumption, may provide important insights for health promotion and disease prevention of immigrants, as they are becoming a large share of the population over the world. Knowledge about dietary habits of both populations groups would contribute to address health disparities, an important public health concern for policy makers and health services.

Therefore, the main purpose of this study is to investigate the adequacy of F&V intake among immigrants living in Portugal, and identify the main determinants of F&V consumption. Additionally, this study extends findings by differentiating the primary outcome between immigrants and Portuguese adult populations.

## 2. Materials and Methods

### 2.1. Population and Sample

**Figure 1:**
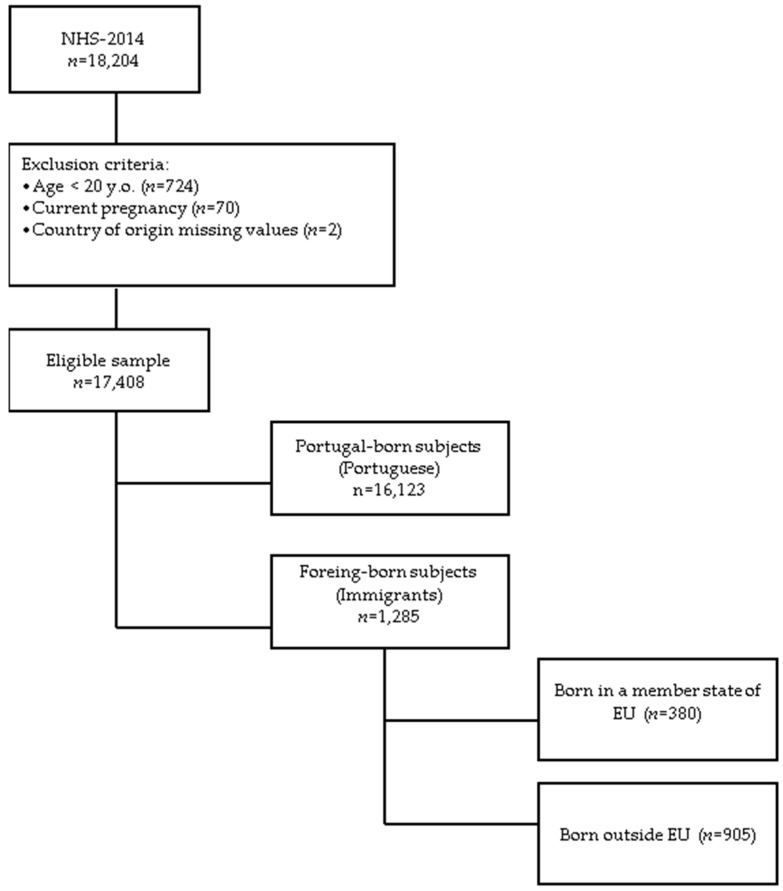
Flowchart for selection of study participants. Legend: NHS: National Health Survey; y.o.: years old; EU: European Union.

Data used in this study come from the National Health Survey (NHS) 2014, the fifth in Portugal since 1987, promoted by the Health Ministry [28]. The NHS 2014 was conducted by the Statistics Portugal Institute (INE) in collaboration with the National Institute of Health Doctor Ricardo Jorge [29]. The microdata database used for the present study was provided under protocol between INE, Directorate-General for Education and Science Statistics (DGEEC), and the Foundation for Science and Technology (FCT). The NHS 2014 is a population-based survey and collected information from the resident population aged 15 and over, on self-assessment health status, health care, and determinants related to lifestyles. This survey followed methodological guidelines and practices regulated at EU level (Commission Regulation (EU) No 141/2013), in order to enable an international comparison of the results. National questions were also included, with a view to obtaining data on relevant issues for characterising the population health status (namely reproductive health, food consumption, satisfaction with life, and long-term disability). INE carried out for the first time the collection of data on the Internet, from a sample survey of households and individuals. Results from the NHS 2014 correspond to estimates of residents, regardless of their nationality or migrant status, in the whole country between September and December 2014. This population-based survey included a total of 22,538 family housing units and the interview completion rate was 80.8% nationwide. The main methodological and conceptual features used in the NHS 2014 have been previously described elsewhere [30]. This study was reported according to the STROBE checklist for observational studies in epidemiology (Supplementary file 1) [31]. Country of birth was used to determine immigrant population and only the adult population, aged 20 and over, was consider for the present study. The final sample size comprised 17,410 participants of whom 7.4% were immigrants (Figure 1).

### 2.2. Variables

The outcome variable of the present study was five or more daily servings of fruit and vegetables (F&V ≥ 5). The formulation of consumption of F&V questions followed the guidelines established by the European Health Interview Survey (EHIS wave 2) Methodological manual [32]. Participants were asked to report the frequency of F&V intake, separately: “once or more a day”; “4 to 6 times a week”; “1 to 3 times a week”; “less than once a week”; or “never”. The question concerning the consumption of fruit was “How often do you eat fruit, including juices squeezed from fresh fruit, and excluding juice made from concentrate? Canned and dried fruit are excluded.” For vegetables intake the question was “How often do you eat vegetables or salad, excluding potatoes and juice made from concentrate? Juices squeezed from fresh or canned vegetables, as well as legumes (beans, lentils), soups (warm and cold), and vegetarian dishes are included.” Those who revealed a daily frequency, “one or more times a day”, were invited to estimate the number of servings per day. One serving was defined as more or less one handful for fruits and three or four tablespoons of vegetables. A show card of examples of fruit, vegetables, and standard portions were used. Ultimately, these variables were joined together to create a dummy variable for adequate F&V consumption.

Sociodemographic (sex, age, education level, legal marital status, job status, urbanisation degree, country of origin, and length of residence), anthropometric (being overweight), and lifestyle (physical activity, smoking, and alcohol habits) characteristics were assessed in the present study as covariates variables.

Education level was divided into three categories: none or basic, secondary, and higher education. Basic education consists of 9 years of school, starting at age six; secondary education spans 3 years, for students aged 15 to 18 years old; and higher education includes post-secondary-level technological specialised courses and university or polytechnic education. Legal marital status was recorded as married or not married. Job status was divided into two categories: employed and not employed. The employed category includes those participants who worked for family without a salary, those with temporary medical incapacity for work or parental leave, and those in vocational training, apprenticeships, or paid traineeships. The “not employed” category includes unemployed, pensioners, students, housewives, permanently incapacitated, unpaid internship or other status. The degree of urbanisation of place of residence was classified on three types of areas: densely populated area (the same as cities or large urban area); intermediate density area (the same as towns and suburbs or small urban area); and thinly-populated area (alternative name: rural area).

The country of origin was not available in database used for the present study. Instead, immigrants were grouped in two categories: inside EU (born in another member state of the European Union) and outside EU (born in another country outside the European Union). Length of residence in Portugal was categorised as follows, 1 to 4; 5 to 9; 10 to 14; and 15 or more years. Similar categories were used in previous research among immigrants in Portugal [33]. Due to simple size constraints the first two categories were amalgamated resulting in only three categories.

Body mass index (BMI) was calculated based on self-reported height and weight data, and for statistical analyses overweight classification was used as a dummy variable (0 = <25.0 kg/m^2^; 1 = ≥25.0 kg/m^2^) [34]. Lifestyle variables included in this study were smoking habits (current smoker if smoke daily or occasionally; never/former if never smoked or stopped smoking); alcohol drinking classes (regular drinking if drink alcohol every day or once or twice a week; light drinkers if consume alcohol one to three times a month; and occasional drinkers if did not consume last year or less than once a month); and physical activity (active and sedentary were assumed if engaged/not engaged in at least 150 minutes a week of any leisure time or sports exercises, respectively).

### 2.3. Statistical Analysis

The statistical analyses included univariate, bivariate, and multivariate analysis. The Chi-square test was carried out to assess differences in proportions for sociodemographic, anthropometric, and lifestyle characteristics between immigrants and natives and between men and women from each group. Using adequate recommended F&V intake (1 if it is adequate or 0 if otherwise) as a dependent variable, we estimated the unadjusted and adjusted odds ratio (OR) using logistic regression. The model was estimated using sample weights provided by the NHS2014 and included all relevant covariates, namely sex, age, education level, legal marital status, job status, urbanisation degree, country of origin, length of residence, BMI, physical activity, smoking status, and drinking classes. Separate models were estimated for men and women. We considered a 5% confidence level and 95% confidence interval (CI). The data analysis was performed using IBM SPSS® Statistics for Windows, version 23.0 (IBM Corp., Armonk, NY, USA).

## 3. Results

Sociodemographic, anthropometric, and lifestyle characteristics of the Portuguese and immigrant populations are described in Table 1. Among the immigrant population, 74.9% were born outside the EU and the median length of residence was 28 years.

Compared with natives, immigrants were younger (68.0% (95% CI: 65.4–70.6) vs. 47.7% (95% CI: 46.9–48.5) being 20 to 49 years old), and more immigrants than Portuguese have attained higher education (29.4% (95% CI: 26.9–31.9) vs. 18.4% (95% CI: 17.8–19.0)). A greater proportion of foreign-born than natives were employed (62.2% (95% CI: 59.5–64.9) vs. 48.6% (95% CI: 47.8–49.4)) and lived in more densely populated areas (56.6% (95% CI: 53.9–59.3) vs. 42.2% (95% CI: 41.4–43.0) lived in cities or urban areas). In both groups, more men than women were overweight, whose prevalence was higher in the Portuguese population (54.4% (95% CI: 53.6–55.2)).

Adequate F&V intake was more prevalent among immigrants (21.1% (95% CI: 19.0–23.4)) than natives (18.5% (95% CI: 17.9–19.1)). Consuming five or more servings of F&V on a daily basis were more frequent among women, in both population groups.

**Figure 2:**
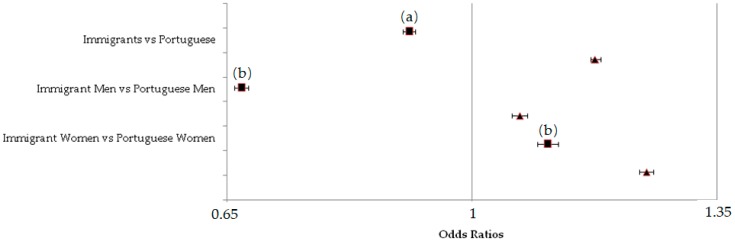
Adjusted (■) and unadjusted (▲) association of migrant status with adequate F&V intake. Legend: (**a**) adjusted for sex, age, educational level, legal marital status, job status, urbanisation degree, body mass index, physical activity, smoking status, and drinking classes; (**b**) adjusted for age, educational level, legal marital status, job status, urbanisation degree, body mass index, physical activity, smoking status, and drinking classes.

Results of the association between adequate F&V intake and migrant status, and after adjustment for all relevant variables (Model 1) are shown in Figure 2.

For Model 1, unadjusted results revealed that being an immigrant was positively associated with adequate F&V intake (OR = 1.18, 95% CI = 1.17–1.18), especially for women (OR = 1.25, 95% CI = 1.24–1.26). However, for the adjusted model, association between migrant status and recommended F&V consumption was only evident for men: immigrants were less likely to achieve an adequate fruit and vegetable intake (OR = 0.67, 95% CI = 0.66–0.68), when compared to Portuguese (Figure 2).

The Model 2 logistic regression (Table 2) considers only the subsample of the immigrant population; the dependent variable is adequate F&V consumption and results are disaggregated by sex.

For the subsample of immigrants (model 2), adequate F&V intake (≥5 servings/ day) was significantly associated with sex, age, and education level, after adjustment for all covariates. Women had 3.6-fold higher odds (95% CI = 3.54–3.70) of having an adequate F&V consumption, than men (Table 2). Immigrants between 35 and 39 years old had 3.3-fold higher odds (OR = 3.35, 95% CI = 3.26–3.45) of achieving F&V recommendations, compared to younger immigrants. Higher education level appears to be a protective factor for adequate F&V intake (OR = 2.44, 95% CI = 2.37–2.51). In both sexes, for the adjusted model, adequate F&V intake was also positively associated with a low urbanisation degree (thinly vs. densely: OR = 1.76, 95% CI = 1.71–1.81). Compared to immigrants from another member state of the EU, immigrants from outside the EU had lower odds of achieving F&V recommendations (OR = 0.64, 95% CI = 0.63–0.66). Time living in Portugal was found to be a risk factor for adequate F&V consumption, after adjustment for all covariates (15 or more years vs. 0–9 years: OR = 0.52, 95% CI = 0.50–0.53). Being overweight (OR = 0.59, 95% CI = 0.58–0.61), a current smoker (OR = 0.46, 95% CI = 0.45–0.47), and an occasional alcohol drinker (occasional vs. regular: OR = 0.63, 95% CI = 0.61–0.64) decrease the odds of adequate F&V. An active lifestyle among immigrants, compared to sedentary behaviour, increased the odds of meeting the recommendations for these two food groups (OR = 1.43, 95% CI = 1.40–1.46).

Stratified by sex, the results showed that the determinants of adequate F&V intake are different among immigrant men and women (Table 2). In men, being employed (OR = 1.76, 95% CI = 1.68–1.85), living in a thinly urbanised area (OR = 3.73, 95% CI = 3.56–3.91), and engaging in an active lifestyle (OR = 1.5, 95% CI = 1.44–1.55) were more likely to consume adequate F&V (Table 2). Length of residence was negatively associated with adequate F&V intake, after adjustment for other factors, especially for men immigrants living in Portugal for 10 to 14 years (OR = 0.05, 95% CI = 0.05–0.05). For long term immigrants (15 or more years) the odds of adequate F&V consumption was 73% lower for men (OR = 0.27, 95% CI = 0.26–0.29), but not significant for women (OR = 1.03, 95% CI = 0.98–1.09) compared to those arriving in Portugal less than 10 years ago. Among women, those who have attained a higher education level were 3.7 times more likely to achieve the WHO recommendation for F&V consumption (OR = 3.66, 95% CI = 3.52–3.81), compared with their counterparts who had none or only basic education.

## 4. Discussion

The main objectives of this study were to investigate the adequacy of F&V intake among immigrants living in Portugal, and identify the main determinants of consumption. Only 21% of immigrants reported an adequate consumption of ≥5 F&V on a daily basis. Among immigrants, sex, age, education, place of residence, and country of origin were found to be important determinants of a healthy diet.

Among immigrants, women were more likely to attain daily adequate servings of F&V compared with men, after adjustment for all covariates. This finding is in line with the work of Volken and colleagues, who studied immigrants in Switzerland and found that women exhibited a lower risk of an inadequate F&V consumption [35]. Kumar and colleagues showed evidence that women reported a higher intake of this food group in all groups of migrants [36]. Sex-differences in food consumption (vegetables, fruit, and berries) suggested that women had a healthier food intake among Russian, Somali, and Kurdish immigrants in Finland [37]. Despite these results, female migrants were, traditionally, at higher risk of the negative effects of migration and dietary acculturation [13]. A work review on changes in food habits among immigrant women showed that a busier lifestyle, higher level of stress, low socialisation, children’s preferences, unavailability of traditional foods, food insecurity, and taste may result in low consumption of F&V and other unfavourable dietary changes that can in turn cause chronic diseases [13]. However, this negative impact seems to be more evident in USA and Canada, whereas in Europe, studies showed less minor negative or even positive impacts. It is possible that in Europe, and particularly in Portugal, those social and environmental factors identifying as having a negative impact in adequate F&V consumption may not be present among immigrant women, or have a minor expression.

In this study, older age and lower urbanisation degree were found to be positively associated with recommended F&V consumption, for both sexes and after adjustment for all variables. Health reasons, cooking skills, and lack of time were found to be some of the age-related factors that may influence dietary habits [24]. It is possible that older immigrants had more awareness of F&V health benefits, more cooking skills and more available time to spend preparing dishes with these food groups. Among immigrants in Switzerland, the relative risk of low F&V intake decreased with age, but no significant association was found between specific dwelling zones (rural/urban) and low or medium F&V intake, compared with the recommendations [35]. Urbanisation may have a positive influence on availability and diversity of fruits and vegetables, but seasonality and prices determine access [26]. In low- and middle-income countries, living in urban areas, alongside with increasing income have been associated to a higher intake of F&V, in part because of the high cost and limited access to fresh-food markets and stores in rural areas [38]. In the present study, immigrants living in rural areas were more likely to consume the recommended daily servings of F&V, compared with those living in densely urban areas. The inhabitants of rural areas in Portugal traditionally have a small area devoted to the production of fresh vegetables and fruits for their own consumption and to share with neighbours. It is possible that immigrants who choose less densely urbanised places to live adopt the traditional growing of one’s own produce. Because the majority of immigrants in the present study were employed, lack of time to achieve and to prepare meals may explain disparities in F&V consumption, for those living in densely urban areas.

In our study, after adjustment for all covariates, factors that were positively associated with adequate F&V intake, in the general immigrant population, revealed different directions when data was disaggregated by sex. Among immigrant women, education was positively associated with adequate daily F&V consumption. Among immigrant men, being employed and engaged in a more active lifestyle increased the odds of attaining recommendations of F&V. These results highlight the need for different gender-based approaches to changing eating habits, for men and women.

Length of residence has been controversially associated with F&V intake. In Canada, the longer South Asians lived in the host country, the greater their consumption of nonstarchy vegetables, but no significant association was found with the fruit group [39]. In the United Kingdom, a study conducted, also with South Asians, found no changes in F&V intake, between recent (≤5 years) and long-term immigrants (>10 years) [40]. A systematic review of the relationship between acculturation and diet, among Latinos in USA, concluded that total F&V consumption were negatively associated with acculturation [41]. Findings from our study were in line with these results. Length of residence may not fully capture the acculturation process and data from age at arrival could add new insight for this discussion, however no such data was available in the database disposable for the present study. Notwithstanding, length of residence has been used as a measure of acculturation in many studies [15,16,42].

Data from the country of origin was not available for the present study. Instead, countries of birth were aggregated into two large groups, with a great heterogeneity among them with regard to sociodemographic factors. This constitutes a limitation to understand how the bond to country of origin and more traditional foodways may influence F&V adequacy. Nevertheless, in this study, results showed that immigrants from outside EU member states were less likely to attain F&V recommendations.

In Portugal, by 2014, two of the main immigrant groups of non-EU origin came from Brazil and China: Brazilians were the main foreign resident community in Portugal (22.1%), and Chinese, although representing only 5.4% of the immigrant population, had a numerical increment of 14.8% over the prior year [43]. These two countries faced a rapid shift in diet, physical activity and morbidity over the past decades [44,45]. This phenomenon is known as “nutrition transition” and is negatively linked with nutrition-related noncommunicable diseases, because of the reduction of F&V consumption, and the reduction of physical activity in work and leisure [46]. By 2011, fruit supply in Brazil and vegetable supply in China were higher than in Europe [47]. Notwithstanding, only 23.6% of the adult Brazilian population consumed the recommended daily F&V servings [48], and the Chinese diet shifted from a traditional diet abundant with vegetables, to a more Westernised dietary pattern, with low F&V consumption, especially in the low-income groups [47,49].

Furthermore, after adjustment for all covariates, immigrant’s poor lifestyle habits (sedentary and current smoker) and overweight were associated with inadequate F&V consumption. Regarding alcohol drinking habits, findings from the present study were consistent with international research, which show that men drink alcoholic beverages more regularly than women [50]. It has been suggested in the literature that wine drinkers tended to consume more F&V, while preference for other alcoholic beverages (beer and spirits) was associate with less healthy dietary habits [51]. Portugal is a wine producer country and consuming this beverage during meals is an acceptable social behaviour, which may explain that drinking occasionally appears to be a risk factor for inadequate F&V intake, for both sexes and compared to regular drinkers. However, for men, but not for women, light drinking seems to be a protector factor for F&V adequacy. On the one hand, those immigrants who are engaged in poor health habits, like drinking, may try to compensate unhealthy outcomes by improving diet behaviour. On the other hand, regular alcohol consumption is traditionally attributed to men and a reduction in the frequency of alcohol drinking, from regular to light, may indicate greater health vigilance and consequently a greater quality dietary improvement. Among women, being a light drinker may indicate a different pattern of alcohol consumption, with regard to the alcoholic beverage preference and the absolute alcohol intake, which have been associated in previous research with dietary pattern [51,52]. It is possible that for women who do not drink alcohol beverages on a regular basis, alcoholic consumption takes place outside meals, and may be a substitute for meals or food choices. Additionally, we cannot exclude under-reporting of intake, especially from those who know to have unhealthy dietary habits. Differential patterns of alcohol consumption between men and women and its effects on diet suffer influenced by biological, social and cultural factors, which are not part of the scope of this study [53]. It would be valuable to future research with immigrant population, to consider additional alcohol exposure variables besides frequency, namely type of alcohol beverages and absolute consumption. It is important to highlight that an inadequate diet in combination with other poor lifestyle choices may increase the risk of immigrants’ unhealthy outcomes and constitute a serious burden on the health services.

Another purpose of the present study was to verify disparities in adequate F&V consumption between natives and immigrants. Results showed a healthier F&V consumption among immigrants than Portuguese, although for men, being an immigrant was negatively associated with F&V≥5 intake. One possible explanation for differences in F&V consumption between natives and immigrants is that they may have better nutritional knowledge and at least equal F&V accessibility and availability than Portuguese. Immigrants were younger, well-educated, and most of them were employed and inhabitants of densely urban areas, which may indicate a low level of food insecurity. Despite that this factor has not been evaluated in the present study, it is known that the predominant barriers to F&V consumption include inaccessibility, cost, and low income [24,27]. Additionally, participants from the NHS were selected from accommodation units and so the sample may potentially favour well-integrated and well-educated immigrants, and not consider vulnerable groups with different socioeconomic characteristics. Comparisons between immigrant and native groups in previous studies revealed mixed results. The Canadian Community Health Survey showed two different outcomes: data from 2009 revealed that immigrants consumed less F&V than those born in Canada [54], while data from 2007 found no statistically significant difference in F&V intake between immigrants and Canadians [55]. In two other studies conducted in the Netherlands, results showed that South Asian (Surinamese and Pakistanis) generally reported lower frequency of fruit and vegetables compared with the Dutch population [56,57]. The Oslo Immigrant Health Profile from 2008, showed that Vietnamese men had the lowest and Turkish women the highest consumption of F&V, while Norwegians were neither the end nor the beginning of spectrum [36]. Conflicting results may be explained by the use of different methodologies to define and measure F&V, and because of the variety of subjects’ ethnic and social backgrounds used in the studies. Literature shows strong evidence that in European countries there are considerable differences between traditional dietary habits among ethnic populations [12].

Findings from the present study revealed that the majority of both Portuguese and immigrant populations do not likely to attain the recommendations of WHO for an adequate F&V consumption. These results are particularly curious because Portugal, as a South European country, was expected to have a food pattern closer to the Mediterranean style, which is characterised by high consumption of fruit and vegetables [58,59]. However, according to the Portuguese Food Balance Sheet (BAP), an analytical instrument of statistical nature that allows the portrait and trend of food availability, Portugal has been progressively moving away from the Mediterranean food standard. Notwithstanding some improvements in the last decade, F&V availability still does not reach the recommendations [60].

Some limitations of the present study should be considered. First, the measuring of F&V intake was based on memory, thus recall problems are involved. Second, NHS included fruit juices and legumes, which is controversial [61,62]. Pure fruit juices can provide most of the nutrient substances that are present in the original fruit, but with less fibre and sometimes with added sugar [62]. Although legumes, often termed beans or pulses, share some bioactive compounds and fibre with vegetables, they are also a protein source, unlike vegetables. In Europe, recent studies have not included legumes when analysing vegetable consumption [63]. Another limitation was social desirability, which may be considered when F&V intake is assessed [62]. Although not within the scope of this study, disparities in the definition of fruit and vegetable food groups, measures and units were observed between studies and make comparisons difficult. Cultural-based perceptions on food groups and portions may influence reported consumption because they may not coincide with the host country’s classification [63]. In order to minimise methodological differences, standardised approaches and tools to collect and quantify information should be developed [61]. Another limitation is that, because part of the information used for this study was collected via computer-assisted web interviewing, some individuals, without internet or with lower computer literacy, may have been excluded. Additionally, we had to merge two classes from the length of residence variable, for sample size constraints, in order to estimate OR and have better precision. Despite this limitation, is our view that the categories of length of residence used in this study are more suitable for comparisons with other studies. Finally, future studies should consider vulnerable groups, namely refugees or illegal immigrants, with different socioeconomic characteristics and health outcomes.

## 5. Conclusions

This study showed that the WHO recommendation of five or more portions of F&V, on a daily basis, was attained by only 21.1% of immigrants and 18.5% of Portuguese population. Despite of this immigrant status, especially among women, increased the odds of achieve the WHO recommendations. Among immigrants, sex, age, education, and place of residence were found to be important determinants of a healthy diet.

The findings from this study on adequacy and disparities of F&V intake, of both native and foreign-born groups of the Portuguese community, highlight the need for strategies to increase F&V consumption for both communities and provide a starting point for more robust investigations. The main strength of the present study is to contribute with knowledge about the main drivers of a healthy diet among immigrants. Effective gender-based strategies to attain recommendations of daily F&V intake should be considered, and particular attention should be given for immigrant men. Future works should explore the role of the urbanisation degree of the place of residence as an important driver for healthy food consumption. More effective measures of dietary acculturation and food insecurity should be included in future studies about health of immigrants.

## Figures and Tables

**Table 1:** Characteristics of Portuguese and immigrant adult study population living in Portugal.

	Portuguese			Immigrants		
Variables	T % (95% CI)	M % (95% CI)	F % (95% CI)	T % (95% CI)	M % (95% CI)	F % (95% CI)
	*n* = 16,123	*n* = 7037	*n* = 9086	*n* = 1285	*n* = 539	*n* = 746
Age (years)						
20–30	21.1 (20.4–25.9)	22.6 (21.6–23.6)	19.9 (19.1–21.7)	22.5 (20.2–24.8)	25.8 (22.1–29.5)	19.8 (16.9–22.7)
35–49	26.6 (25.9–27.3)	27.7 (26.7–28.7)	25.5 (24.6–26.4)	45.5 (42.8–48.2)	44.0 (39.8–41.2)	46.8 (43.2–50.4)
50–64	25.5 (24.8–26.2)	25.7 (24.7–26.7)	25.3 (24.4–26.2)	23.4 (21.1–25.7)	24.8 (21.2–28.4)	22.1 (19.1–25.1)
65+	26.8 (26.1–27.5)	24.0 (23.0–25.0)	29.3 (28.4–30.2)	8.6 (7.1–10.1)	5.4 (3.5–7.3)	11.3 (9.0–13.6)
*p* value	0.000 *	0.000 **			0.000 **	
Education level						
None or basic	64.4 (63.7–65.1)	65.6 (64.5–66.7)	63.4 (62.4–64.4)	38.9 (36.2–41.6)	40.0 (35.9–44.1)	37.9 (34.4–41.4)
Secondary	17.2 (16.6–17.8)	18.4 (17.5–19.3)	16.2 (15.4–17.0)	31.7 (29.2–34.2)	34.4 (30.4–38.4)	29.4 (26.1–32.7)
Higher education	18.4 (17.8–19.0)	16.0 (15.1–16.9)	20.4 (19.6–21.2)	29.4 (26.9–31.9)	25.6 (21.9–29.3)	32.6 (29.2–36.0)
*p* value	0.000 *	0.000 **			0.000 **	
Legal marital status						
Married	60.3 (59.5–61.1)	64.0 (62.9–65.1)	56.9 (55.9–57.9)	54.9 (52.2–57.6)	55.7 (51.5–59.9)	54.2 (50.6–57.8)
*p* value	0.000 *	0.000 **			0.000 **	
Job status						
Employed	48.6 (47.8–49.4)	54.0 (52.8–55.2)	43.9 (42.9–44.9)	62.2 (59.5–64.9)	69.4 (65.5–73.3)	56.0 (52.4–59.6)
*p* value	0.000 *	0.000 **			0.000 **	
Urbanisation degree						
Densely	42.2 (41.4–43.0)	41.4 (40.2–42.6)	42.9 (41.9–43.9)	56.6 (53.9–59.3)	54.8 (50.6–59.0)	58.1 (54.6–61.6)
Intermediate	30.1 (29.4–30.8)	30.2 (29.1–31.3)	30.0 (29.1–30.9)	24.8 (22.4–27.2)	27.5 (23.7–31.3)	22.6 (19.6–25.6)
Thinly	27.7 (27.0–28.4)	28.4 (27.3–29.5)	27.1 (26.2–28.0)	18.6 (16.5–20.7)	17.7 (14.5–20.9)	19.3 (16.5–22.1)
*p* value	0.000 *	0.000 **			0.000 **	
Anthropometric						
Overweight	54.4 (53.6–55.2)	58.8 (58.0–60.0)	50.6 (50.0–51.6)	47.4 (44.7–50.1)	51.3 (47.1–55.5)	44.1 (40.5–47.7)
*p* value	0.000 *	0.000 **			0.000 **	
F&V intake						
≥5 servings/day	18.5 (17.9–19.1)	15.7 (14.8–16.5)	21.0 (20.2–21.8)	21.1 (19.0–23.4)	16.6 (13.5–19.7)	25.0 (21.9–28.1)
*p* value	0.000 *	0.000 **			0.000 **	
Physical activity						
Active	63.4 (62.7–64.1)	65.8 (66.9–69.1)	60.5 (59.5–61.5)	62.8 (60.2–65.4)	64.5 (60.5–68.5)	60.5 (57.0–64.0)
*p* value	0.000 *	0.000 **			0.000 **	
Smoke status						
Current	20.3 (19.7–20.9)	28.8 (27.7–29.9)	12.8 (12.1–13.5)	23.7 (21.4–26.0)	28.8 (25.0–32.6)	19.4 (16.6–22.2)
*p* value	0.000 *	0.000 **			0.000 **	
Drinking classes						
Regular	46.1 (45.3–46.9)	68.0 (66.9–69.1)	26.9 (26.0–27.8)	39.9 (37.2–42.6)	57.5 (53.3–61.7)	24.8 (21.7–27.9)
Light	13.8 (13.3–14.3)	12.6 (11.8–13.4)	14.8 (14.1–15.5)	18.5 (16.4–20.6)	16.3 (13.2–19.4)	20.4 (17.5–23.3)
Occasional	40.1 (39.3–40.9)	19.4 (18.5–20.3)	58.3 (57.3–59.3)	41.6 (38.9–44.3)	26.2 (22.5–29.9)	54.7 (51.1–58.3)
*p* value	0.000 *	0.000			0.000	

*p* value from chi-square tests; T: Total; M: Male; F: Female; CI: Confidence intervals; *: differences in proportions between populations; **: differences in proportions between sexes.

**Table 2:** Correlates of adequate F&V consumption for the subsample of 1285 immigrants: logistic regression results.

	F&V ≥ 5 Servings per Day					
Variables	Total		Men		Women	
	Unadjusted	Adjusted †	Unadjusted	Adjusted ‡	Unadjusted	Adjusted ‡
Sociodemographic						
Sex						
Men	1	1	1	1	1	1
Women	1.67 (1.65–1.69) *	3.62 (3.54–3.70) *	n.a	n.a	n.a	n.a
Age						
20–34y	1	1	1	1	1	1
35–39y	2.50 (2.46–2.55) *	3.35 (3.26–3.45) *	2.70 (2.62–2.77) *	3.44 (3.27–3.62) *	2.25 (2.20–2.31) *	4.44 (4.26–4.63) *
50–64y	1.81 (1.77–1.85) *	2.00 (1.93–2.08) *	1.10 (1.93–2.06) *	3.20 (3.00–3.41) *	1.65 (1.60–1.69) *	2.02 (1.93–2.12) *
65+ y	3.60 (3.51–3.69) *	8.10 (7.75–8.47) *	4.83 (4.64–5.04) *	21.19 (19.63–22.87) *	2.73 (2.65–2.81) *	5.69 (5.34–6.06) *
Education level						
None or basic	1	1	1	1	1	1
High school	1.32 (1.30–1.34) *	1.94 (1.88–1.99) *	1.49 (1.46–1.53) *	1.62 (1.56–1.69) *	1.25 (1.22–1.27) *	1.61 (1.55–1.68) *
Higher educ.	2.19 (2.16–2.22) *	2.44 (2.37–2.51) *	2.64 (2.58–2.71) *	0.99 (0.95–1.03)	1.88 (1.85–1.92) *	3.66 (3.52–3.81) *
Legal marital status						
Not married	1	1	1	1	1	1
Married	1.16 (1.15–1.18) *	0.61 (0.60–0.63) *	1.28 (1.26–1.31) *	0.78 (0.75–0.81) *	1.11 (1.09–1.13) *	0.48 (0.46–0.49) *
Job status						
Not Employed	1	1	1	1	1	1
Employed	1.24 (1.23–1.26) *	1.28 (1.25–1.31) *	1.79 (1.75–1.84) *	1.76 (1.68–1.85) *	1.17 (1.15–1.19) *	0.91 (0.88–0.94) *
Urbanisation degree						
Densely	1	1	1	1	1	1
Intermediate	0.59 (0.58–0.60) *	1.67 (1.63–1.71) *	1.66 (1.62–1.70) *	2.34 (2.25–2.42) *	0.98 (0.97–1.00)	1.59 (1.54–1.65) *
Thinly	0.69 (0.68–0.70) *	1.76 (1.71–1.81) *	2.51 (2.45–2.58) *	3.73 (3.56–3.91) *	1.33 (1.31–1.36) *	1.22 (1.18–1.26) *
Country of origin						
Inside EU	1	1	1	1	1	1
Outside EU	0.69 (0.68–0.70) *	0.79 (0.77–0.81) *	0.57 (0.56–0.58) *	0.67 (0.64–0.69) *	0.79 (0.77–0.80) *	0.73 (0.71–0.75) *
Length of residence						
0–9 years	1	1	1	1	1	1
10–14 years	1.23 (1.10–1.15) *	0.20 (0.19–0.21) *	1.00 (0.97–1.04)	0.05 (0.05–0.05) *	1.27 (1.23–1.31) *	0.46 (0.43–0.49) *
15+ years	1.32 (1.29–1.34) *	0.52 (0.50–0.53) *	0.72 (0.70–0.74) *	0.27 (0.26–0.29) *	2.10 (2.04–2.16) *	1.03 (0.98–1.09)
Anthropometric						
Body mass index						
<25 kg/m2	1	1	1	1	1	1
≥25 kg/m2	0.83 (0.82–0.84) *	0.59 (0.58–0.61) *	1.05 (1.03–1.07) *	0.64 (0.62–0.66) *	0.75 (0.73–0.76) *	0.62 (0.61–0.64) *
Lifestyle						
Physical activity						
Sedentary	1	1	1	1	1	1
Active	1.24 (1.22–1.26) *	1.43 (1.40–1.46) *	1.72 (1.67–1.78) *	1.50 (1.44–1.55) *	1.11 (1.08–1.14) *	1.13 (1.10–1.16) *
Smoking status						
Never/ former	1	1	1	1	1	1
Current	0.49 (0.48–0.50) *	0.46 (0.45–0.47) *	0.36 (0.35–0.37) *	0.19 (0.18–0.20) *	0.67 (0.66–0.69) *	0.72 (0.69–0.74) *
Drinking classes						
Regular	1	1	1	1	1	1
Light	1.12 (1.10–1.14) *	0.70 (0.68–0.72) *	1.12 (1.09–1.15) *	1.42 (1.36–1.48) *	0.90 (0.88–0.92) *	0.57 (0.55–0.59) *
Occasional	0.99 (0.97–1.00)	0.63 (0.61–0.64) *	0.67 (0.66–0.69) *	0.48 (0.46–0.50) *	0.84 (0.83–0.86) *	0.81 (0.78–0.84) *

OR: Odds Ratio; CI: Confidence Intervals; 1: reference group; n.a: not applicable; * *p* < 0.05. † Adjusted for sex, age, education level, legal marital status, job status, urbanisation degree, country of origin, length of residence, body mass index, physical activity, smoking status, and drinking classes. ‡ Adjusted for age, education level, legal marital status, job status, urbanisation degree, country of origin, length of residence, body mass index, physical activity, smoking status, and drinking classes.

## Supplementary Materials

The following are available online at http://www.mdpi.com/1660-4601/15/10/2299/s1, File S1: STROBE Statement—Checklist of items that should be included in reports of cross-sectional studies.
